# Inhomogeneity of epidemic spreading with entropy-based infected clusters

**DOI:** 10.1063/1.4824316

**Published:** 2013-10-10

**Authors:** Zhou Wen-Jie, Wang Xing-Yuan

**Affiliations:** 1 Faculty of Electronic Information and Electrical Engineering, Dalian University of Technology, Dalian 116024, China; 2 School of Electronic and Information Engineering, Lanzhou Jiaotong University, Lanzhou 730070, China

## Abstract

Considering the difference in the sizes of the infected clusters in the dynamic complex networks, the normalized entropy based on infected clusters ($\delta^{*}$) is proposed to characterize the inhomogeneity of epidemic spreading. $\delta^{*}$ gives information on the variability of the infected clusters in the system. We investigate the variation in the inhomogeneity of the distribution of the epidemic with the absolute velocity *v* of moving agent, the infection density *ρ*, and the interaction radius *r*. By comparing $\delta^{*}$ in the dynamic networks with $\delta_{H}^{*}$ in homogeneous mode, the simulation experiments show that the inhomogeneity of epidemic spreading becomes smaller with the increase of *v*, *ρ*, *r*.

The epidemic spreading in human populations is a typical public health incident, and has also become one of the major public safety issues that humanity is facing until now. In order to conveniently study the epidemic spreading, researchers usually adopt the homogeneous mixing hypotheses. However, such a hypothesis is not strong. Recently, researchers have discussed the credibility of the hypothesis. In this paper, we further study this credibility and carry out simulation experiments in the dynamic network. The results indicate that the inhomogeneity of epidemic spreading is affected by the absolute velocity *v*, the infection density ***ρ***, and the interaction radius *r*.

## I. INTRODUCTION

The studied models of the epidemic spreading have greatly improved the public's understanding of infection mechanisms.^1–6^ Among the most studied models for investigating the spread of epidemic, susceptible-infected-susceptible (SIS) and susceptible-infected-recovered (SIR) epidemic models are two famous epidemiological models.^7,8^ When studying the epidemic spreading with these models in complex networks, researchers often adopt homogeneous mixing hypotheses^9–12^ which each infected agent has same probability of contact with any susceptible agent. However, such a hypothesis is not strong and should be thoroughly discussed. Recently, researchers have discussed the credibility of the hypothesis. For instance, Liu *et al.* implement the distribution of an epidemic is always inhomogeneous after the transition process in Refs. 13 and 14, where they present the characteristic infected cluster size (CICS), which characterizes the inhomogeneity of the epidemic spreading. However, the CICS measurement studied on the typical size of the largest infected cluster, which cannot reflect the infected clusters.

Entropy is a fundamental concept in physics. It is related to the information content and order/disorder of a system. In statistical physics, cluster entropy has been used in the study of problems, such as percolation^15,16^ and complex systems. Cluster entropy was utilized within the Axelrod model to measures the number of cultural groups of different sizes.^17^ Cluster-size entropy was also used in the Axelrod's cultural adaptation model which gives information on the variability of the cultural cluster size present in the system.^18^ In the paper, the normalized entropy based on infected clusters ($\delta^{*}$) can reflect the variability of the infected clusters in dynamic model. In addition in order to characterize the inhomogeneity of the epidemic spreading, we denote the Homogeneous Mode^13^ where all the infected agents are randomly distributed and keep static in the system, is used to represent the case where the epidemic is homogeneously distributed. For the convenience of discussion, we denote the $\delta^{*}$ of homogeneous mode as $\delta_{H}^{*}$. Therefore, for given infection density *ρ*, the larger $\left|\delta^{*}-\delta_{H}^{*}\right|$ is, the more inhomogeneous the epidemic spreading is.

## II. THE DYNAMIC MODEL

In order to be closer to reality, we consider the case that agents may travel by transport to long distance.^19,20^ So, in this paper the agents not only may perform the local motion but also move into long position. It is assumed that agents *M* are distributed randomly in a square of linear size *L* with periodic boundary conditions initially. Each agent can jump into any position (i.e., long position) inside the square with the probability *q* or perform the local motion with the probability 1−q. Considering the fact that the jumping process is much shorter than the infection period, we assume that the status of the jumping agents will be kept when it move. At each time step Δt, the local motion process is modeled as follows:

$$\left\{ \begin{array}{c} x_{i}(t+\Delta t)=x_{i}(t)+\vec{v_{i}}(t)\Delta t\\ \theta_{i}(t)=\xi_{i}, \end{array}\right.$$ (1)

where $x_{i}(t)$ is the position of the agent *i* at time *t*. $\vec{v_{i}}(t)$ is characterized by *v* and $\theta_{i}(t)$. The absolute velocity *v* of all the agents is the same and remains constant in motion. $\theta_{i}(t)$ is motion direction of the agent *i* at time *t* and $\xi_{i}$ follows the uniform distribution in the interval [0,2π).

In the model, each agent can be in two distinct states: susceptible (S) or infected (I). Some agents are infected at the seed of the infection, and all the others are susceptible. And from then on, their positions and states are updated at each time step. Concretely, if the agent *i* is infected currently, then it is cured and becomes susceptible at the next time step with probability *μ*; if it is susceptible currently, then it can be infected with probability $1-{\left(1-\alpha \right)}^{k(i)}$ at t time where α is the infection rate and k(i) is the infected neighbors set of the agent *i*. Here k(i) is defined as the infected agents in the spherical neighborhood of the interaction radius *r* centered on the agent *i*

$$k(i)=\left\{j|\left\|x_{j}-x_{i}\right\|\leq r,j\in M,j\neq i\right\},$$ (2)

where ‖•‖ denotes the Euclidean distance between *i* and *j* in two-dimensional space. If $\left\|x_{j}-x_{i}\right\|\leq r$, j≠i, then *i* and *j* are neighbors and can infect each other.^21,22^ And we assume that each agent has the same interaction radius. Without lack of generality, let us set μ=1 since it only affects the time scale of the infection evolution. Here, the above spread mechanism will run until the system reaches its stationary state or the epidemic dies out.

## III. ENTROPY-BASED INFECTED CLUSTERS

In the dynamic model, each node represents an agent and each link represents a connection along which an epidemic can spread. The cluster is the subnet whose nodes are connected^20,22^ (i.e., from any node one can reach any other node along links in the subnet), the infected cluster only includes infected agents. The infected cluster size is the number of infected agents of the infected cluster. Let $C=\{c_{1},c_{2},\ldots ,c_{k}\}$ be the set of the infected clusters in the network. In this study, entropy-based infected clusters is defined as

$$\delta =-\sum_{1\leq i\leq \left|C\right|}p(i)\ln p(i),$$ (3)

where p(i) is the probability that an infect agent belongs to $c_{i}$ of *C*. We can calculate p(i) by

$$p(i)=\frac{\left|c_{i}\right|}{\sum_{j}\left|c_{j}\right|},$$ (4)

obviously, $\delta_{\max}$, which denotes the maximum value of δ, is equal to ln(m), when p(i)=1/m for each 1≤i≤m, where *m* is the number of the infected agents in the dynamic network. Similarly, the minimum value of δ for the dynamic network with *m* infected agents, denoted as $\delta_{\min}$, is equal to 0, which is obtained when the infected agents gather into one cluster.

The normalized entropy based on infected clusters is defined as

$$\delta^{*}=\frac{\delta -\delta_{\min}}{\delta_{\max}-\delta_{\min}}=\frac{\delta }{\ln m}.$$ (5)

From Eq. (5), when $\delta^{*}$ is approaching to 0, the infected agents will converge into a single cluster. When $\delta^{*}$ is approaching to 1, the infected agents will show a scattered distribution and the formed clusters appear with a similar size.

## IV. RESULTS AND DISCUSSIONS

In this section, we will discuss the inhomogeneity of the epidemic spreading while the agents walk randomly in the square (v>0), and suppose v∈[0.1,10]. In all the simulation experiments, *L* is set to 10 and the number of agents *M* is fixed to 200. The initial proportions of the infected agents are 10%, while all the others start from the susceptible state. *m* is the number of infected agents. And then we update agents' state synchronously in the model for Δt (Δt is set to 1) time steps. All data points shown in each figure are acquired by averaging over 50 runs.

**FIG. 1.**
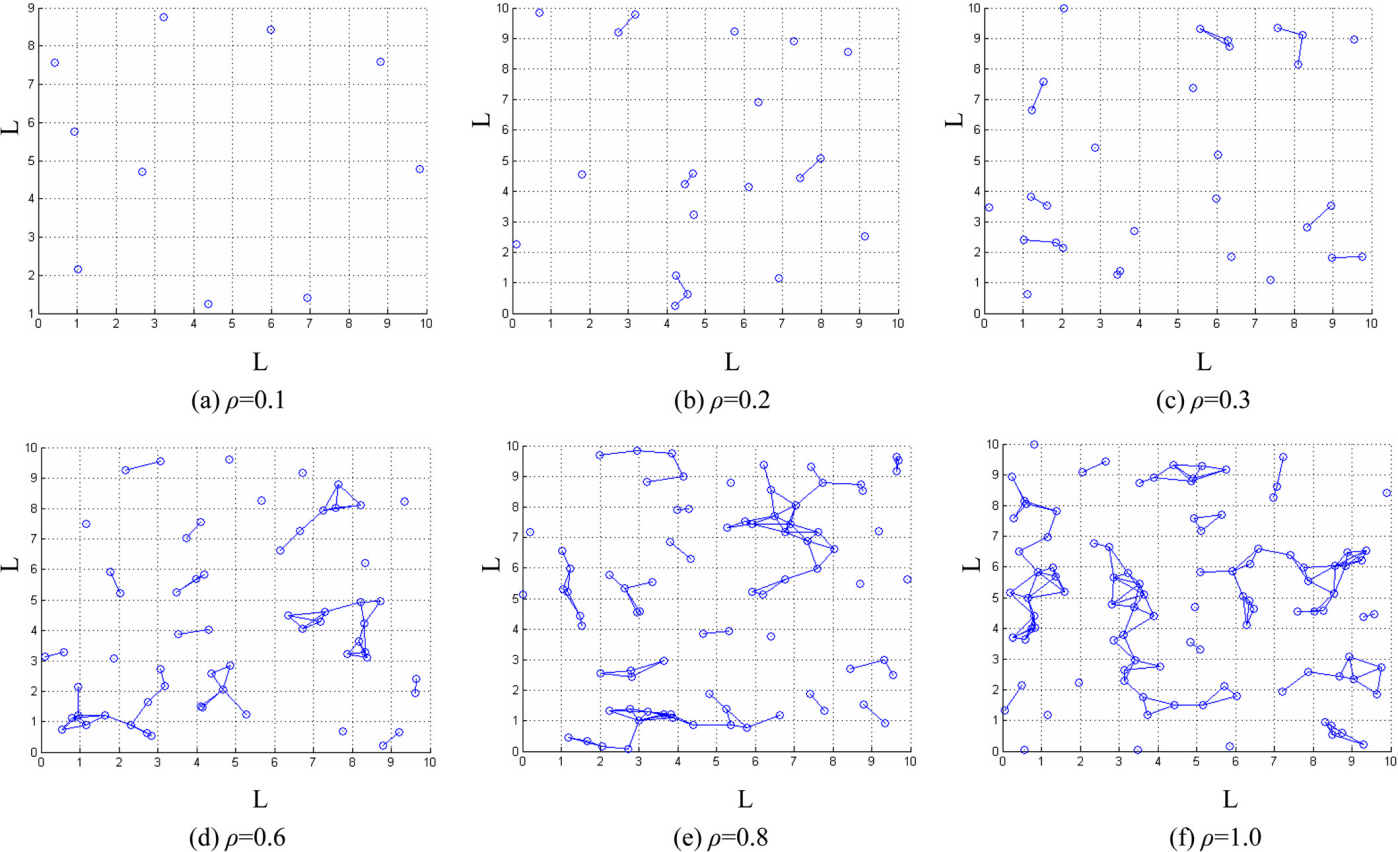
The distribution of infected clusters in different infection density.

In Fig. 1, one can find that the distributions of the infected clusters are different when the infection density $\rho =m/L^{2}$ is different and r=1. When *ρ* is low, the infected agents can scatter small clusters with similar size as shown in Figs. 1(a)–1(c). When *ρ* is high, infected agents are prone to gather into large clusters in Figs. 1(d)–1(f). The CICS is cannot reflect the variability of the infected clusters in the system. However, $\delta^{*}$ can give information on the variability of the infected clusters. Next, we will discuss the inhomogeneity of the epidemic spreading by a comparable analysis on $\delta^{*}$ in the dynamic networks and $\delta_{H}^{*}$ in homogeneous mode.

**FIG. 2.**
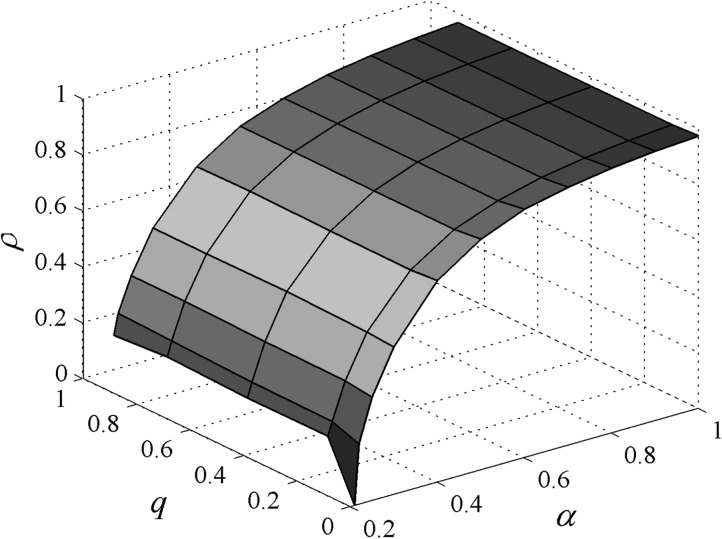
*ρ* versus *q* and *α*.

The moving of agents cannot affect the proportion of the infected agents in Ref. 13. Fig. 2 shows the combined effect of *q* and α on the evolution of *ρ* for r=1, v=0.5. For a fixed *v*, one can find that *ρ* increases monotonously with α. The reason for this is that when the infection rate α is large, the probability that a susceptible agent is infected by its infected neighbors is also high. Thus, more susceptible agents get infected. In addition, the result of numerical simulation shows that *ρ* is close to 1 with the increase of α and *q*.

**FIG. 3.**
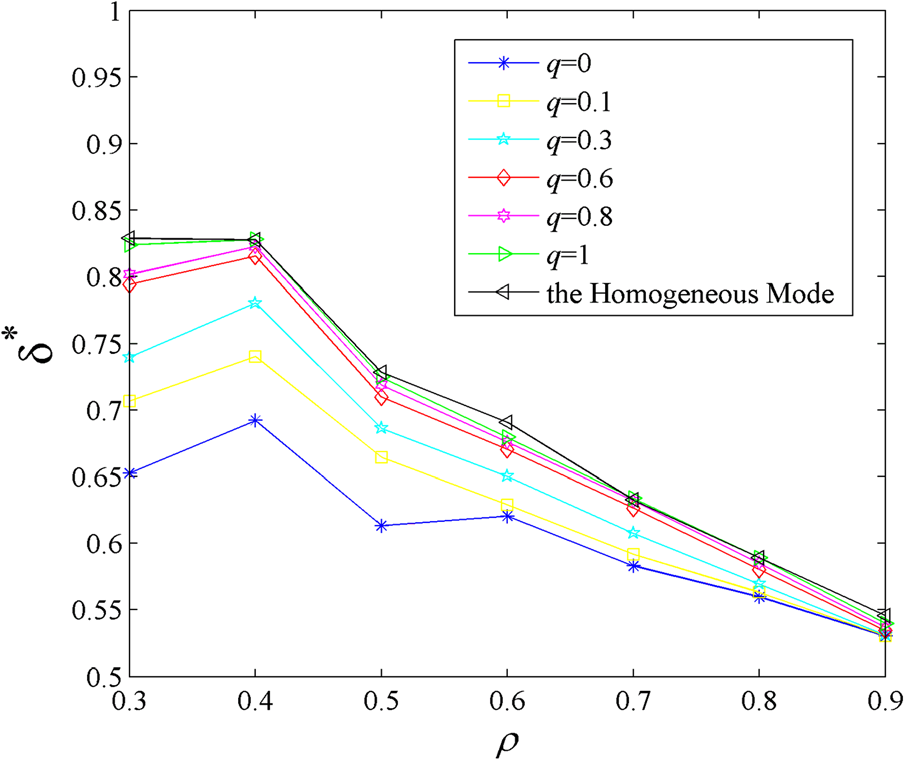
$\delta^{*}$ versus *ρ*, with the change of *q*.

When r=1, v=0.5, we can plot $\delta^{*}$ versus *ρ* with different *q* (as illustrated in Fig. 3). The comparable curve in homogeneous mode ($\delta_{H}^{*}$) is also given in Fig. 3 with black color. It shows that $\delta^{*}$ is close to $\delta_{H}^{*}$ when q→1, which it means the infected agents are distributed homogeneously. Obviously, this conclusion is coincident with that of theoretical analysis. However, $\delta^{*}$ is apparently less than $\delta_{H}^{*}$ when q∈[0,0.6) and ρ∈[0.2,0.7] which it means the epidemic spreading is inhomogeneous. This conclusion by letting q=0 is coincident with that of previous results.^13^

**FIG. 4.**
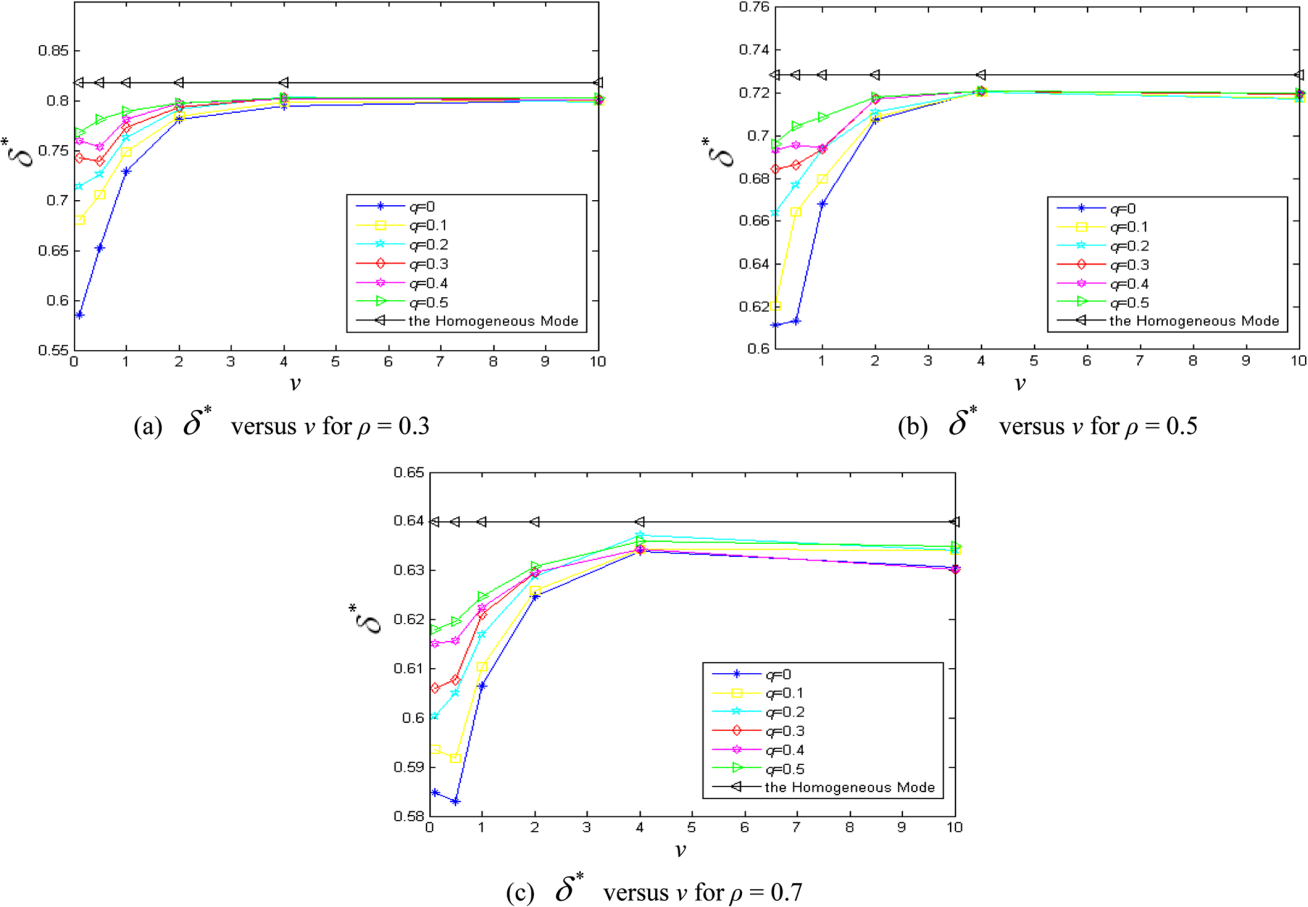
$\delta^{*}$distribution for different values of *v*.

To study the effect of the absolute velocity *v* on the inhomogeneity of the epidemic spreading when ρ∈[0.2,0.7], q∈[0,0.6) and r=1, Fig. 4 demonstrates the $\delta^{*}$ as a function of *v* for different values of *q* with ρ=0.15, 0.25, and 0.35, respectively. To comparison, in Fig. 4(a) we plot six curves (v=0.3, 0.1, 0.5, 1, 2, 4, 10, respectively) of dynamic network and a straight line ($\delta_{H}^{*}$) of homogeneous mode when ρ=0.3. Similarly, Figs. 4(b) and 4(c) show the situations when ρ=0.5, 0.7, respectively. One can find that the variation of the inhomogeneity is related to *v*. When v∈(0.1,2], $\delta^{*}$ is apparently less than $\delta_{H}^{*}$ for each *ρ*. That means, the epidemic spreads inhomogeneously. When v>2, $\delta^{*}$ is close to $\delta_{H}^{*}$ for each *ρ*. Namely, the epidemic spreading is homogeneous. In fact, when the agents move with a high velocity, they have greater chance to jump out of the area that is covered by its infected neighbors than that in the case of small *v*. Therefore, the infected agents cannot gather into a large cluster. In addition, The infected density *ρ* affect the difference between the maximum and minimum of $\delta^{*}$. When ρ=0.3 and v=0.1 (or v=2)

$$\delta_{\max}-\delta_{\min}=0.22(0.05),$$

when ρ=0.7 and v=0.1 (or v=2)

$$\delta_{\max}-\delta_{\min}=0.02(0.01).$$

Then we can say that the smaller is the infected density *ρ*, the stronger *v* affects the inhomogeneity of the epidemic spreading. And the finding is in accordance with the previous work.^13^

**FIG. 5.**
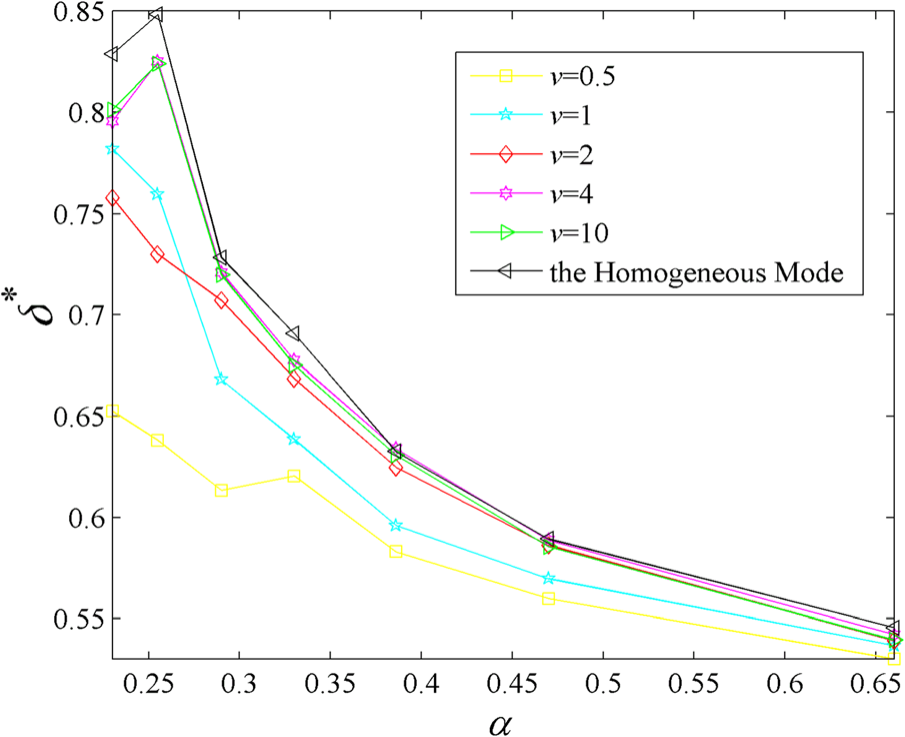
$\delta^{*}$ versus α with the change of *v*.

**FIG. 6.**
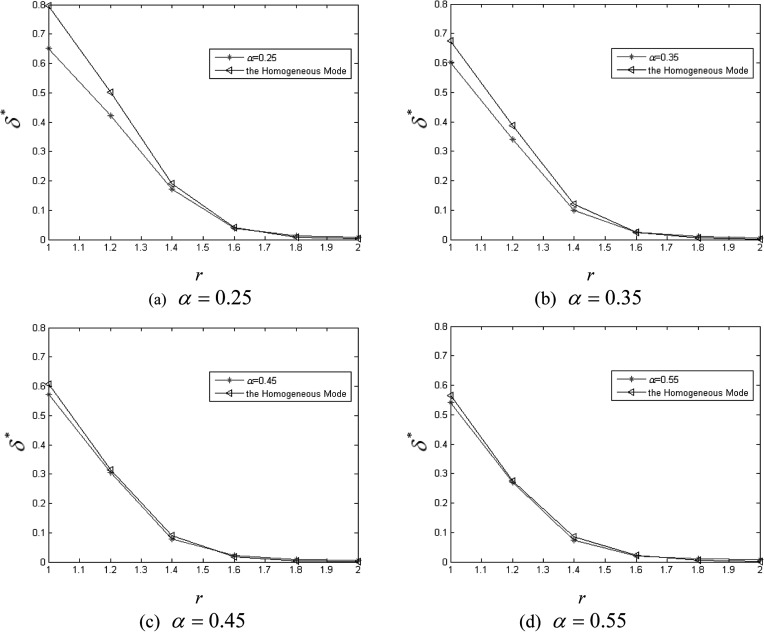
$\delta^{*}$ versus *r* for different values of *α*.

Previous results^13,14^ have not discussed about the role of the interaction radius *r* on the inhomogeneity of the epidemic spreading. Fig. 5 presents $\delta^{*}$ as a function of α for various *v* when r=1, q=0. One can find the epidemic spreads inhomogeneously when v≤2, α<0.6. Next, suppose r∈[1,2], we plot $\delta^{*}$ against *r* for different values of α when q=0, v=0.5 in Fig. 6. As can be seen in Fig. 6, $\delta^{*}$ is approaching to $\delta_{H}^{*}$ with the increase of *r* which it means that the epidemic spreading tends to a homogeneous state. Our results indicate the inhomogeneity decreases even disappears with the increase of *r*. And one can see that $\delta^{*}$ monotonously decreases with the increase of *r*. The reason for this is that the neighbors can infect each other in interaction neighborhoods which are determined by the radius *r* at each time step. As pairwise interactions increase with *r*, it is possible for a susceptible agent to be infected more by its infected neighbors. So the infected agents are prone to form large infected clusters.

## V. CONCLUSIONS

In this paper, we investigate inhomogeneity of the epidemic spreading by the normalized entropy based on infected clusters ($\delta^{*}$) in the dynamic network. $\delta^{*}$ can reflect the variability of the infected clusters in the system. The simulations show that $\delta^{*}$ decreases with the increase of the infection density *ρ* and the interaction radius *r*. That means, the infected agents prone to form large clusters as the infected agents always infect their neighbors. And the inhomogeneity of epidemic spreading decreases with the increase of *v*, *r*.

## References

[1] B. T. Grenfell, O. N. Bjornstad, and J. Kappey, “ Travelling waves and spatial hierarchies in measles epidemics,” Nature 414(6865), 716–723 (2001). 10.1038/414716a 11742391

[2] X. P. Han, “ Disease spreading with epidemic alert on small-world networks,” Phys. Lett. A 365(1), 1–5 (2007). 10.1016/j.physleta.2006.12.054

[3] L. M. Sander, C. P. Warren, I. M. Sokolov, C. Simon, and J. Koopman, “ Percolation on heterogeneous networks as a model for epidemics,” Math. Biosci. 180, 293–305 (2002). 10.1016/S0025-5564(02)00117-7 12387929

[4] K. B. Blyuss, “ On a model of spatial spread of epidemics with long-distance travel,” Phys. Lett. A 345, 129–136 (2005). 10.1016/j.physleta.2005.07.004

[5] X. Li, Y. Y. Jin, and G. Chen, “ Complexity and synchronization of the word trade web,” Physica A 328, 287–296 (2003). 10.1016/S0378-4371(03)00567-3

[6] A. James, J. W. Pitchford, and M. J. Plank, “ An event-based model of superspreading in epidemics,” Proc. R. Soc. Ser. B 274(1610), 741–747 (2007). 10.1098/rspb.2006.0219 PMC2197209 17255000

[7] W. O. Kermack and A. G. McKendrick, “ Contributions to the mathematical theory of epidemics,” Proc. Roy. Soc. London Ser. A 115(5), 700–721 (1927). 10.1098/rspa.1927.0118

[8] H. W. Hethcote, “ The mathematics of infectious diseases,” SIAM Rev. 42(4), 599–653 (2000). 10.1137/S0036144500371907

[9] M. Boguñá and R. Pastor-Satorras, “ Epidemic spreading in correlated complex networks,” Phys. Rev. E 66(4), 047104 (2002). 10.1103/PhysRevE.66.047104 12443385

[10] M. Barthélemy, A. Barrat, R. Pastor-Satorras, and A. Vespignani, “ Velocity and hierarchical spread of epidemic outbreaks in scale-free networks,” Phys. Rev. Lett. 92(17), 178701 (2004). 10.1103/PhysRevLett.92.178701 15169200

[11] J. Zhou, Z. Liu, and B. Li, “ Influence of network structure on rumor propagation,” Phys. Lett. A 368(6), 458–463 (2007). 10.1016/j.physleta.2007.01.094

[12] C. Xia, S. Sun, Z. Liu, and Z. Chen, “ Influence of mobile agents on the spreading behavior of SIS model,” Phys. Procedia 3(5), 1825–1830 (2010). 10.1016/j.phpro.2010.07.025

[13] Z. Z. Liu, X. Y. Wang, and M. G. Wang, “ Inhomogeneity of epidemic spreading,” Chaos 20(2), 023128 (2010). 10.1063/1.3445630 20590324 PMC7117601

[14] Z. Z. Liu, X. Y. Wang, and M. G. Wang, “ Variations in epidemic distribution with some characteristic parameters,” Chin. Phys. B 21(7), 078901 (2012). 10.1088/1674-1056/21/7/078901

[15] I. J. Tsang, I. R. Tsang, and D. Van Dyck, “ Cluster diversity and entropy on the percolation model: The lattice animal identification algorithm,” Phys. Rev. E 62(5), 6004–6014 (2000). 10.1103/PhysRevE.62.6004 11101928

[16] I. R. Tsang and I. J. Tsang, “ Cluster size diversity, percolation, and complex systems,” Phys. Rev. E 60(3), 2684–2698 (1999). 10.1103/PhysRevE.60.2684 11970070

[17] J. C. Villegas-Febres and W. Olivares-Rivas, “ The existence of negative absolute temperatures in Axelrod's social influence model,” Physica A 387(14), 3701–3707 (2008). 10.1016/j.physa.2008.02.001

[18] Y. Gandica, A. Charmell, J. Villegas-Febres, and I. Bonalde, “ Cluster-size entropy in the Axelrod model of social influence: Small-world networks and mass media,” Phys. Rev. E 84(4), 046109 (2011). 10.1103/PhysRevE.84.046109 22181229

[19] T. Vicsek, A. Czirok, E. Ben-Jacob, I. Cohen, and O. Shochet, “ Novel type of phase transition in a system of self-driven particles,” Phys. Rev. Lett. 75(6), 1226–1229 (1995). 10.1103/PhysRevLett.75.1226 10060237

[20] A. Buscarino, L. Fortuna, M. Frasca, and A. Rizzo, “ Dynamical network interactions in distributed control of robots,” Chaos 16(1), 015116 (2006). 10.1063/1.2166492 16599782

[21] M. Frasca, A. Buscarino, A. Rizzo, L. Fortuna, and S. Boccaletti, “ Dynamical network model of infective mobile agents,” Phys. Rev. E 74(3), 036110 (2006). 10.1103/PhysRevE.74.036110 17025711

[22] Z. Liu and B. Hu, “ Epidemic spreading in community networks,” Europhys. Lett. 72(2), 315–321 (2005). 10.1209/epl/i2004-10550-5

